# Supplementary material for: Treatment of C3 Glomerulopathy in Adult Kidney Transplant Recipients: A Systematic Review
Source: Med Sci (Basel). 2020 Oct 21;8(4):44. doi: 10.3390/medsci8040044 (PMC7712822; doi:10.3390/medsci8040044)
Supplement: Supplementary file 1 [file medsci-08-00044-s001.pdf]

Search terms for systematic review.

Databases: Ovid MEDLINE

1. Kidney.mp
2. Renal.mp
3. exp kidney/
4. 1 OR 2 OR 3
5. Transplant.mp
6. Transplantation.mp
7. exp transplant/
8. exp transplantation/
9. 5 OR 6 OR 7 OR 8
10. 4 AND 9
11. c3 glomerulopathy.mp
12. exp c3 glomerulopathy/
13. c3 glomerulonephritis.mp
14. exp c3 glomerulonephritis/
15. dense deposit disease.mp
16. exp dense deposit disease.
17. C3G.mp
18. C3GN.mp
19. DDD.mp
20. 11 OR 12 OR 13 OR 14 OR 15 OR 16 OR 17 OR 18 OR 19
21. 10 AND 20

Databases: EMBASE:

('kidney transplantation' OR 'kidney graft' OR 'kidney graft rejection') AND (‘c3 glomerulopathy' OR 'c3 glomerulonephritis ' OR ‘dense deposit disease ')

Database: Cochrane Databases

('kidney transplantation' OR 'kidney graft' OR 'kidney graft rejection') AND (‘c3 glomerulopathy' OR 'c3 glomerulonephritis ' OR ‘dense deposit disease ') in Title Abstract Keyword'
